# 

**Published:** 1890-12

**Authors:** 


### CONTENTS—DECEMBER, 1890.—VOL. VI., NO. 6.

ORIGINAL ARTICLES— PAGE.
Fracture of the Head of Humerus, Exsection, etc. A. N. Denton, M. D. .... 231
Hermaphrodite [?] in Insane Asylum. F. S. White, M. D. .... 236
Appendicitis. A. S. McDaniel, M. D. .... 238
Complete Rupture of the Perineum, etc. W. R. Blailock, M. D. .... 241
Tubercular Peritonitis with Ascites following Measles—Treatment. L. H. Luce, M. D. .... 245
Indieations for Operation in Ectopic Gestation. C. A. L. Reed, M. D. .... 246
A Remarkable case of Desquamation. L. W. Skipper, M. D. .... 249
SOCIETY NOTES—
West Texas Medical Association .... 250
Cherokee County Medical Society .... 251
Note from Dr. Howeth, President North Texas Medical Society .... 252
ITEMS OF INTEREST—
Therapeutics, etc. .... 253
EDITORIAL—
Dr. Ghent's Hepatic Pills .... 257
Koch's "Cure" .... 259
Asylum Superintendents .... 265
MEDICAL NEWS AND MISCELLANY—
Marriages, Deaths, Removals, etc., of Doctors, Therapeutics, Prescriptions, etc. .... 261
BOOK NOTICES—
Post-Mortems .... 273
PUBLISHER'S NOTES—
Containing Valuable Information .... 274

